# Our Experience in the Management of Splenic Arterial Thrombosis

**DOI:** 10.7759/cureus.105161

**Published:** 2026-03-13

**Authors:** Gia Tomadze, Avto Megreladze, Gia Azmaiparashvili, Giorgi Danelia, Ilona Kurashvili

**Affiliations:** 1 Surgery, Pineo Clinic, Tbilisi, GEO; 2 Surgery, Tbilisi State Medical University, Tbilisi, GEO; 3 General Surgery, Tbilisi State Medical University, Tbilisi, GEO

**Keywords:** abdominal surgery, anticoagulation treatment, arterial thrombosis, spleen abscess, spleen thrombosis, splenectomy

## Abstract

This article presents a combined literature overview and a three-case clinical series highlighting the diagnostic complexity and therapeutic decision-making in splenic arterial and venous thrombosis. Although splenic infarction is a rare cause of acute abdominal pain, the article emphasizes its high clinical significance and its association with systemic thromboembolic disease, atrial fibrillation, hematologic disorders, hypercoagulable states, pancreatitis, and splanchnic venous thrombosis.

The literature summarized by the authors underscores the modern shift toward non-operative management in hemodynamically stable patients without rupture or infectious complications. Contemporary multicenter data demonstrate a substantial survival benefit from anticoagulation in splenic infarction, without an associated increase in bleeding risk. Conversely, surgery is now reserved for complicated cases, including splenic rupture, abscess formation, or secondary peritonitis. Regarding isolated splenic vein thrombosis, the authors discuss evolving evidence showing high rates of spontaneous or anticoagulation-related recanalization and low rates of variceal bleeding, suggesting that routine splenectomy is no longer indicated except in cases complicated by infection.

The article’s three clinical cases exemplify these principles. It is concluded that the decisive factor in treatment selection is not the presence of thrombosis alone, but the development of complications, especially abscess, rupture, sepsis, and peritonitis. In uncomplicated cases, anticoagulation remains safe and effective, while surgery should be used selectively for life-threatening sequelae.

## Introduction

Splenic thrombosis (arterial or venous) most often presents clinically as splenic infarction, a rare but important cause of acute abdominal pain. Large hospital-based series suggest that splenic infarction accounts for only about 0.016% of all admissions, underlining how infrequently this diagnosis is made in general practice [1]. The main etiologic groups are thromboembolic disease (especially atrial fibrillation and cardiogenic emboli) and hematologic or hypercoagulable states (myeloproliferative neoplasms, sickle cell disease, antiphospholipid antibodies, oral contraceptive use, malignancy). In patients younger than 40 years, hematologic and hypercoagulable disorders predominate, whereas in older patients, cardioembolic sources such as atrial fibrillation are more common [2,3]. Importantly, up to one-third of splenic infarctions represent the first manifestation of a previously unrecognized systemic disease, so detection should always prompt a search for an underlying prothrombotic condition.

Splenic vein thrombosis (SVT) is usually discussed within the broader entity of splanchnic vein thrombosis. Acute and chronic pancreatitis are now recognized as leading causes of isolated SVT. In a 2025 single-center series of 426 patients with acute pancreatitis, 10.1% developed splanchnic vein thrombosis; among these, SVT was present in 79.1%, often in combination with portal or mesenteric vein thrombosis [4]. A systematic review and meta-analysis cited in the same article reported an overall prevalence of splanchnic vein thrombosis of 13.6% in pancreatitis, with higher rates in acute than in chronic disease [5]. In cirrhotic patients undergoing splenectomy, portal or SVT is a particularly frequent complication. A meta-analysis of 33 observational studies including 2,997 cirrhotic patients found a pooled prevalence of portal/splenic vein thrombosis of 22.2% after splenectomy, with higher rates in more advanced Child-Pugh classes [5].

Because of this heterogeneity in etiology and the presence of underlying liver or pancreatic disease, management strategies vary. For hemodynamically stable splenic infarction without rupture, current reviews and expert statements favor non-operative treatment: analgesia, hydration, management of the underlying cause, and, when not contraindicated, systemic anticoagulation [2]. A multicenter cohort study from Taiwan, including 86 patients with splenic infarction, showed that 52.3% of patients received anticoagulant therapy. Anticoagulated patients had a 94% relative reduction in all-cause mortality (HR 0.06, 95% CI 0.007-0.48) without an increased risk of major bleeding [1]. Previous series summarized in the same paper found that 30%-100% of patients with splenic infarction receive anticoagulants in routine practice [1].

For splanchnic vein thrombosis in general, guidance documents (e.g., ISTH and other expert statements) recommend anticoagulation for at least 3-6 months in patients with symptomatic acute thrombosis and no active bleeding, aiming for vessel recanalization and prevention of extension or recurrent events [6]. Low-molecular-weight heparin followed by vitamin K antagonists or direct oral anticoagulants is commonly used. In the setting of pancreatitis-related splanchnic thrombosis, observational data suggest that most cases can be managed conservatively, with anticoagulation reserved for progressive or symptomatic thrombosis, and that mortality is usually determined by the underlying pancreatitis rather than by the thrombosis itself [4].

## Case presentation

The article presents three successfully managed cases of splenic thrombosis treated in our clinic. In two cases, non-operative conservative treatment with anticoagulants was sufficient, while in one case, operative treatment (splenectomy) was required.

Case 1

A 78-year-old female patient was admitted to the clinic on January 20, 2025, complaining of pain in the left upper quadrant radiating to the left hypogastrium. The pain had begun two days earlier. Its intensity increased, the abdomen became distended, the pain spread to the entire left half of the abdomen, and general weakness and nausea appeared, because of which she was brought to the clinic by emergency services.

Her medical history included arterial hypertension and atrial fibrillation. She was not receiving anticoagulant therapy. All necessary clinical and diagnostic evaluations were performed.

**Figure 1:**
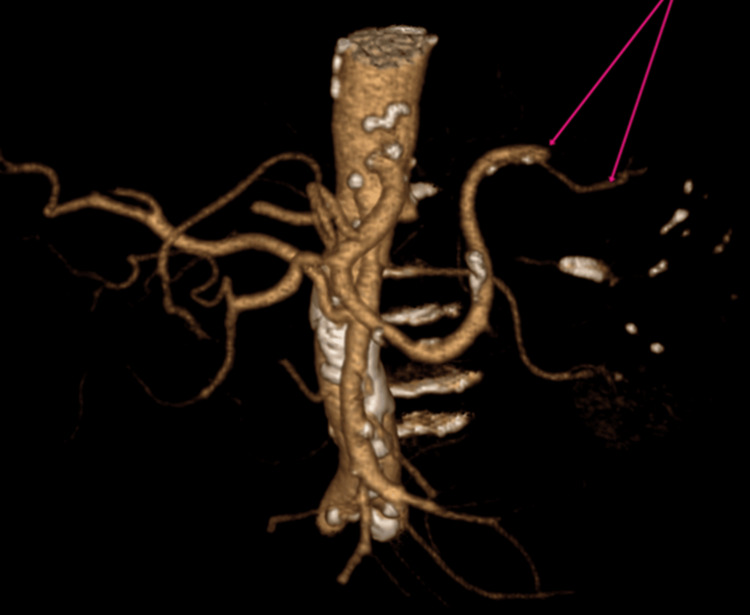
3D-volume-rendered CT angiography demonstrating abrupt cessation of contrast opacification in the distal splenic artery (pink arrows), indicating complete thrombosis. Proximal branches remain patent while distal perfusion is absent, correlating with extensive splenic infarction (Case 1)

An abdominal CT scan with IV contrast revealed total thrombosis of the distal segment of the splenic artery (Figure 1). From the middle third of the artery, a 3-mm collateral vessel originated, which enhanced with contrast and supplied the medial segment of the spleen. Approximately 50% of the spleen showed no contrast enhancement. The corresponding venous branch was also thrombosed. Additionally, the left kidney had two arteries: a 7-mm main artery with total thrombosis, and a 3-mm caudally located artery that was fully contrast-enhanced. As a result, contrast enhancement was seen only in the lower third of the left renal parenchyma.

Laboratory data of the first case indicate the presence of a generalized thrombotic disorder (Table 1). 

**Table 1:** Laboratory results indicating thrombosis WBC: white blood cell, aPTT: activated partial thromboplastin time.

Parameters	Results	Reference range
WBC	18.7 x 10^9^/L	4.0-11.0 x 10⁹/L
Band neutrophils	9%	0%-3%
Segmented neutrophils	70%	40%-60%
Creatinine	202 mmol/L	50-110 mmol/L
D‑dimer	3,030 ng/mL	<500 ng/mL
aPTT	65.10 sec	25-35 sec

The patient was evaluated by an angiologist, urologist, general surgeon, and cardiologist. At this stage, urgent surgical intervention was not considered necessary. She was admitted to the surgical ward for continued treatment and monitoring. Conservative therapy was initiated, including antithrombotic, antibiotic, infusion, spasmolytic, cardiotropic, gastroprotective, and symptomatic treatments, with continuous monitoring. Initially, she received Heparin Belmed (Heparin sodium) 5,000 IU/mL subcutaneously once, after which Enoxaparin 0.8 mL subcutaneously every 12 hours was administered for nine days.

Her general condition gradually improved, pain decreased, and laboratory results returned toward normal. Two follow-up abdominal CT scans with contrast were performed. The CT scan on January 29, 2025, showed that, compared with prior scans, contrast enhancement in the medial splenic segment had become heterogeneous, and a large hypodense area had appeared.

The patient was discharged 10 days after admission. As outpatient therapy, she was prescribed Rivaroxaban 15 mg once daily with food. During nine months of follow-up, her condition remained stable with no significant complications. Thus, in this case, splenic and left renal thrombosis did not require operative intervention and were successfully managed conservatively.

Case 2

A 70-year-old male patient was admitted on March 25, 2024, complaining of a painful mass in the right inguinal region, nausea, vomiting, and general weakness. He had had an inguinal hernia for several years that periodically became incarcerated, but he had previously been able to reduce it himself. Two days earlier, the hernia became incarcerated again, and he was unable to reduce it, prompting him to seek medical care. Two weeks prior, he had been treated for pneumonia. He also had a known hepatic echinococcal cyst for which he had not received treatment, as well as arterial hypertension and atrial fibrillation on ECG.

Immediately upon admission, he underwent inguinal hernioplasty according to Lichtenstein’s technique. On postoperative day 2, he developed abdominal distension and diffuse abdominal pain. Abdominal and urinary tract ultrasound showed the liver, spleen, and other organs without pathology. Bilateral hydroceles were present. A CT scan without angiographic contrast (due to a high creatinine level of 370 µmol/L) showed no major pathology. The following day, repeat CT with angiographic contrast revealed a slight increase in intra-abdominal fluid compared with the previous scan, and separation of the liver’s costal surface by 20 mm. No contrast enhancement of the splenic parenchyma was seen in any phase, raising suspicion of splenic infarction.

**Figure 2:**
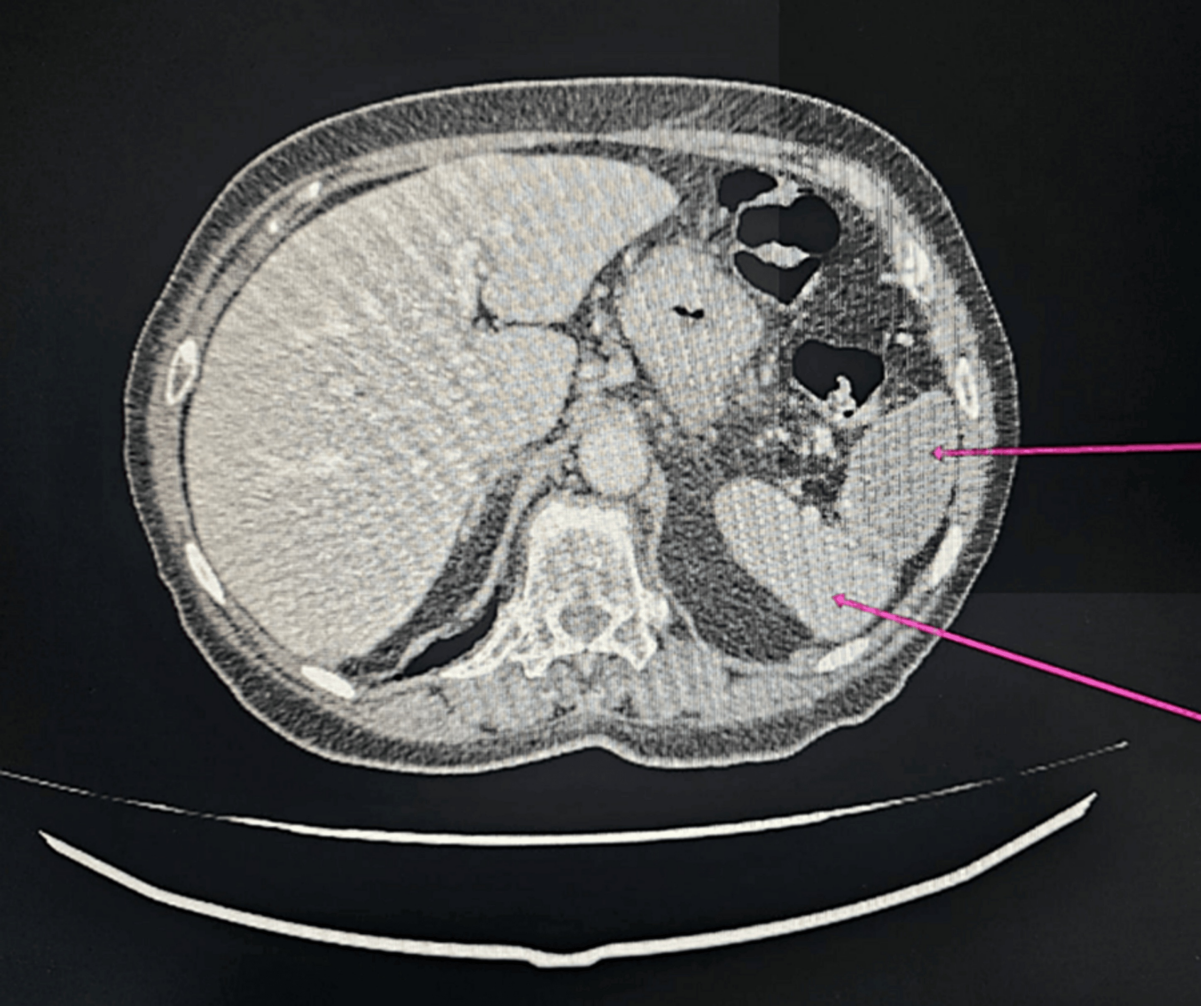
Splenic thrombosis (Case 2)

On the same day (March 28, 2024), scrotal ultrasound demonstrated complete absence of vascularization in the right testis on power Doppler. On postoperative day 3, the patient’s condition worsened: pain and abdominal distension increased, and leukocytosis rose to 34,000. Considering the ultrasound and CT findings, thromboembolic complications were suspected: splenic and right testicular infarction. Emergency laparotomy was performed (Figure 2).

Findings

Approximately 700-800 mL of hemorrhagic fluid was present in the abdominal cavity, extending into both lateral gutters and the recto-vesical pouch. The spleen was markedly enlarged (splenomegaly), measuring 25-28 cm in length and 18 cm in width. Its mid-portion and lower pole were darkened, with a bronze-like mottled appearance. At the mid-level, the capsule had a transverse laceration with a circumferential 4-cm defect filled with clot. The right testis was cyanotic, and ultrasonography confirmed absent vascularization. Splenectomy and right orchiectomy were performed.

The postoperative course was severe. The patient was treated in intensive care for acute renal failure, encephalopathy, and other somatic complications. Anticoagulation initially included Enoxaparin 0.6 mL subcutaneously every 12 hours during the first 24 hours. Subsequently, Heparin Belmed (Heparin sodium) 5,000 IU/mL was administered subcutaneously every 6 hours for 15 days, after which Enoxaparin 0.4 mL every 12 hours was given for 3 days. He was discharged on postoperative day 19. Outpatient anticoagulation included Rivaroxaban 15 mg twice daily for one week, followed by Rivaroxaban 20 mg once daily for long-term therapy.

Thus, in this case, splenic thrombosis and infarction developed on the background of atrial fibrillation and the absence of anticoagulation, requiring operative management.

Case 3

A 69-year-old male patient was admitted on March 13, 2025. He was brought in by family members with complaints of diffuse abdominal pain, distension, and nausea. According to relatives, the symptoms had begun four days prior to hospitalization. His medical history included an ischemic stroke in 2022 with subsequent dementia and episodes of encephalopathy, as well as a myocardial infarction in 2002. He was not receiving any active medical therapy at the time of admission.

**Figure 3:**
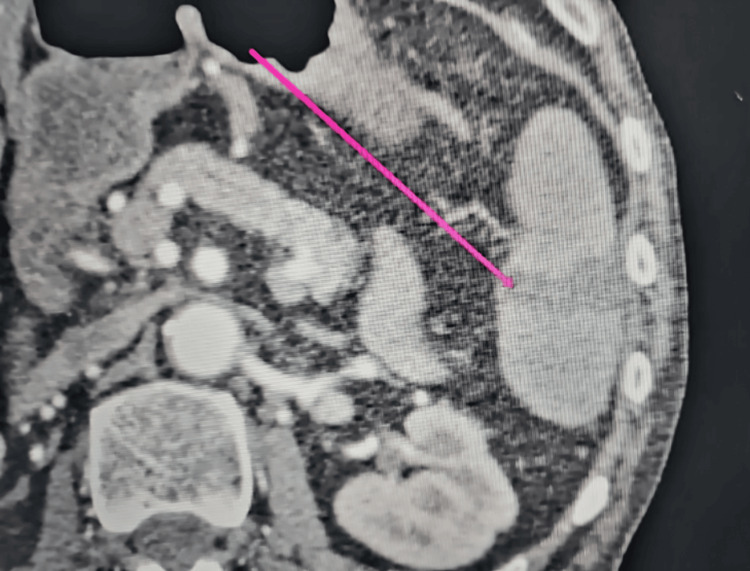
Splenic segmental thrombosis (Case 3)

CT scan demonstrated small‑bowel ileus. Partial thrombosis of the superior mesenteric artery, splenic artery, and segmental renal arteries was observed. The inferior mesenteric artery was completely thrombosed (Figure 3).

Emergency laparotomy was performed. Findings included segmental acute vascular necrosis of the intestine, acute peritonitis, and splenic thrombosis (without significant macroscopic changes). Thromboendarterectomy of the superior mesenteric artery was performed, along with partial small‑bowel resection and entero‑enterostomy.

Two days later, the patient again developed an acute abdomen. Repeat CT showed free air in the abdominal cavity and a small intestinal segment lacking contrast enhancement, suggesting recurrent thrombosis and bowel perforation. Relaparotomy was performed, with resection of 5 cm of necrotic small bowel and creation of a Maydl-type enterostomy. Intensive therapy followed. Anticoagulation included Heparin Belmed (Heparin sodium) 5,000-30,000 IU/mL via pump during the first 24 hours, followed by Enoxaparin 0.8 mL subcutaneously every 12 hours for 11 days.

Upon discharge, outpatient anticoagulation included Nadroparin calcium 0.6 mL once daily subcutaneously for 10 days, Rivaroxaban 15 mg once daily for 6 months, and thereafter Cardiomagnyl (Acetylsalicylic acid + magnesium hydroxide) 75 mg once daily permanently.

Thus, despite severe comorbidities and the need for bowel surgery, splenic thrombosis in this case was also managed conservatively.

## Discussion

The role of surgery in splenic thrombosis is now more restricted. Arterial splenic thrombosis typically results from embolic or in situ arterial occlusion and leads to acute splenic ischemia with wedge-shaped infarction, often presenting with sudden left upper quadrant pain and an increased risk of splenic rupture. In contrast, SVT causes impaired venous outflow, resulting in congestive splenomegaly, parenchymal edema, and potential development of left-sided portal hypertension (LSPH). Its clinical course is usually subacute or chronic and frequently associated with pancreatitis, cirrhosis, or underlying hypercoagulable conditions.

A recent multicenter retrospective study of 98 patients with iSVT found that 39 received anticoagulation and 59 did not. Anticoagulation was associated with a recanalization rate of approximately 46% versus 15% without anticoagulation, while the overall rate of upper GI variceal bleeding was only 3%, and no patient required splenectomy during a 10-year observation period [6]. Historically, splenectomy was recommended as the definitive treatment for LSPH, with gastric variceal bleeding caused by SVT, and contemporary guidelines still consider splenectomy in patients with recurrent or life-threatening upper GI bleeding clearly attributable to SVT [6]. However, modern series emphasize that many patients can be treated endoscopically and with anticoagulation alone, and that routine prophylactic splenectomy for asymptomatic iSVT is not supported by current evidence [6].

Splenic thrombosis represents a challenging vascular complication most commonly associated with acute and chronic pancreatitis, pancreatic pseudocysts, pancreatic malignancy, and local inflammatory processes involving the splenic hilum [4,7,8]. According to multiple contemporary series, the incidence of SVT in acute pancreatitis ranges between 7% and 20%, while in chronic pancreatitis, it may reach 20%-40%, particularly in the presence of peripancreatic collections and pseudocysts [4,8]. The pathophysiology involves local inflammation, direct venous compression, activation of the coagulation cascade, and endothelial injury, ultimately resulting in impaired venous outflow from the spleen and the development of LSPH [7,8].

Clinically significant variceal bleeding occurs in a minority of patients, reported between 4% and 12% across large cohorts, yet the mortality of uncontrolled gastric variceal hemorrhage remains substantial [7-9]. Several studies emphasize that most patients with SVT remain asymptomatic, and the diagnosis is often incidental during CT or MR angiography performed for pancreatitis evaluation [8-10]. Nonetheless, the presence of splenomegaly, gastric varices, or unexplained anemia should raise suspicion for SVT and possible LSPH [9,11].

The role of anticoagulation in isolated SVT remains controversial. Retrospective analyses demonstrate recanalization in up to 30%-45% of patients receiving anticoagulation, compared with 15%-20% spontaneous recanalization without therapy [6,7]. However, the risk of exacerbating variceal bleeding limits routine anticoagulation in patients with established gastric varices [7,11]. Recent meta-analyses conclude that anticoagulation should be individualized: it is indicated in cases of propagating thrombosis, extensive splanchnic thrombosis associated with acute pancreatitis, or hypercoagulable states, but used cautiously when varices are present [6,12,13].

Splenectomy has historically been considered the definitive treatment for symptomatic SVT, especially in the presence of recurrent gastric variceal bleeding. Reported bleeding control after splenectomy approaches 90%-100%, with very low recurrence [12]. Current surgical literature supports splenectomy for patients with significant LSPH manifestations or those with life-threatening hemorrhage [11,12]. Conversely, in asymptomatic SVT discovered incidentally, splenectomy is not routinely recommended [9,11]. Emerging endovascular approaches, such as splenic artery embolization or variceal obliteration, are increasingly reported as less invasive alternatives in high-risk surgical candidates [11,13].

The management of SVT associated with pancreatic surgery or pancreatic malignancy represents a separate clinical scenario. In patients undergoing distal pancreatectomy, splenic vein ligation may predispose to segmental portal hypertension. Large surgical series report postoperative varices in up to 40% of cases, although clinically significant bleeding remains uncommon [14]. Prophylactic splenectomy during elective pancreatic surgery is not recommended; instead, individualized risk assessment and postoperative surveillance are advised [11,14].

Recent publications highlight the prognostic role of SVT in necrotizing pancreatitis, where extensive splanchnic thrombosis may correlate with higher rates of infected necrosis, organ failure, and need for intervention [10,14]. Nevertheless, isolated SVT without extension into the portal or superior mesenteric veins does not typically worsen overall prognosis [6,10].

Splenic infarction may result from several mechanisms, including cardioembolic events (particularly in patients with atrial fibrillation), sepsis-associated thrombosis due to systemic inflammatory response and hypercoagulability, arterial thrombosis secondary to atherosclerosis or local vascular injury, venous thrombosis (SVT), hypercoagulable states including malignancy and inflammatory conditions, and hemodynamic instability, especially prolonged hypotension [15-17].

In our case, although the patient had documented atrial fibrillation, the splenic infarction occurred in the context of severe sepsis due to an incarcerated hernia with systemic inflammatory response. Sepsis is known to induce a prothrombotic state through endothelial dysfunction, platelet activation, and cytokine-mediated coagulation cascade activation. Therefore, both AF-related embolism and sepsis-induced thrombosis were considered possible contributors. While atrial fibrillation remains a plausible source of embolism, the temporal relationship with septic shock suggests that sepsis-related hypercoagulability may have played a significant role.

## Conclusions

Our clinical experience with three cases of splenic arterial thrombosis demonstrates that management must be driven by the presence or absence of infectious and inflammatory complications rather than by the thrombosis itself. In one patient, the development of splenic abscess and secondary peritonitis necessitated urgent splenectomy, which provided definitive control of infection and resolved the acute surgical condition. In contrast, the two patients without abscess formation or peritoneal signs were successfully treated conservatively with anticoagulation, achieving stable clinical courses without progression of thrombosis.

These cases highlight that, in the absence of sepsis-related complications, non-operative management is both safe and effective. Surgical intervention should be reserved for patients who develop abscess, peritonitis, or other life-threatening sequelae of splenic thrombosis.
